# ATP6AP2 knockdown in cardiomyocyte deteriorates heart function via compromising autophagic flux and NLRP3 inflammasome activation

**DOI:** 10.1038/s41420-022-00967-w

**Published:** 2022-04-04

**Authors:** Lei Li, Ya-juan Cui, Yu Liu, Hui-xin Li, Yu-dong Su, Sheng-nan Li, Lan-lan Wang, Yue-wen Zhao, Shuang-xi Wang, Feng Yan, Bo Dong

**Affiliations:** 1 Department of Cardiology, Shandong Provincial Hospital, Cheeloo College of Medicine, Shandong University, 250012 Jinan, China; 2 The Key Laboratory of Cardiovascular Remodeling and Function Research, Chinese Ministry of Education, Chinese National Health Commission and Chinese Academy of Medical Sciences, The State and Shandong Province Joint Key Laboratory of Translational Cardiovascular Medicine, Department of Cardiology, Qilu Hospital, Cheeloo College of Medicine, Shandong University, 250012 Jinan, China; 3 Department of Cardiology, Shandong Provincial Hospital, Shandong First Medical University & Shandong Academy of Medical Sciences, 250012 Jinan, China; 4 Shandong University of Traditional Chinese Medicine, 250012 Jinan, China; 5 Department of Emergency Medicine, Qilu Hospital, Cheeloo College of Medicine, Shandong University, 250012 Jinan, China

**Keywords:** Mitophagy, Disease model, Inflammasome, Heart failure

## Abstract

Moderate autophagy can remove damaged proteins and organelles. In some inflammatory diseases, autophagy plays a protective role by inhibiting the NOD-like receptor family pyrin domain containing 3(NLRP3). (Pro)renin receptor (PRR, or ATP6AP2) is a critical component of the V-ATPase required for autophagy. It remains controversial about ATP6AP2 in the pathological process. The impact of ATP6AP2 on NLRP3 inflammasome and autophagic flux remains unknown under pressure overload stress. This research explores the potential link between ATP6AP2, autophagic flux, and NLRP3. There was upregulation of ATP6AP2 from 5-day post-TAC, and this expression remained at a high level until 8-weeks post-TAC in wild mice. Meanwhile, autophagic flux switched from early compensatory activation to blocking in the heart failure phase. NLRP3 activation can be seen at 8-week post-TAC. Adenovirus-mediated knockdown of ATP6AP2(shR-ATP6AP2) accelerated the progress of heart failure. After TAC was induced, shR-ATP6AP2 significantly deteriorated heart function and fibrosis compared with the shR-Scr group. Meanwhile, there was an elevated expression of NLRP3 and autophagic flux blockage. A transgenic mouse(Tg) with cardio-restricted ATP6AP2/(P)RR overexpression was constructed. Although high expression in cardiac tissue, there were no spontaneous functional abnormalities under the basal state. Cardiac function, fibrosis, hypertrophy remained identical to the control TAC group. However, SQSTM1/P62 was reduced, which indicated the relief of autophagic flux blockage. Further, Neonatal rat ventricular myocyte (NRVMs) transfected with shR-ATP6AP2 showed more susceptibility than sh-Scr NRVMs to phenylephrine-induced cell death. More reactive oxygen species (ROS) or mito-ROS accumulated in the shR-ATP6AP2 group when phenylephrine stimulation. Blocking NLRP3 activation in vivo partly rescued cardiac dysfunction and fibrosis. In conclusion, ATP6AP2 upregulation is a compensatory response to pressure overload. If not effectively compensated, it compromises autophagic flux, leads to dysfunctional mitochondria accumulation, further produces ROS to activate NLRP3, eventually accelerates heart failure.

## Introduction

As a leading cause of morbidity and mortality, heart failure (HF) causes an enormous economic impact [1, 2]. Fully understanding the mechanisms would bring bight new therapeutic strategies for the treatment of heart failure.

Autophagy and mitophagy maintain intracellular homeostasis in cardiomyocytes. The loss of function of autophagy-related genes exacerbates pathological progression in myocardial infarction, atherosclerosis [3–5], doxorubicin-induced cardiomyopathy [6]. Nevertheless, some inconsistencies and controversial results remain about the influence of autophagy on remodeling in HF [7–10]. Hence, the exact role of autophagy in cardiac remodeling requires further study.

Emerging evidence has indicated that full activation of the NLRP3 inflammasome and secretion of IL-1β and IL-18 [11] participated in atherosclerosis, diabetes, heart failure [12–14]. Besides, many researchers have found that autophagy and NLRP3 inflammasome activation are mutually regulating processes [15–19]. So maintaining the average autophagic flux is essential for cell survival.

ATP6AP2 was initially found as a fragment related to proton pump V-ATPase, which is pivotal for maintaining the PH gradient and lysosome function in cells. Then, in 2002, ATP6AP2 was found to bind with prorenin and facilitate prorenin processing to renin and was misnamed as (pro)renin receptor (PRR) [20]. Several studies showed that ATP6AP2 participates in the pathogenesis of acute or chronic kidney diseases, hypertension, fibrosis, diabetes, and various other conditions [21–26]. Our previous research showed that ATP6AP2/PRR has high expression and participation in fibrosis in diabetic cardiomyopathy and alcoholic cardiomyopathy [25, 27]. However, other studies failed to confirm a relationship between high ATP6AP2 overexpression and pathological function [28, 29]. There were no spontaneous cardiac morphological abnormalities in Atp6ap2 transgenic overexpressed mice at baseline. Cardiac hypertrophy and cardiac or renal fibrosis did not show any differences between Tg and wild mice after isoproterenol infusion for 28 days. However, research involving genetic manipulation of ATP6AP2 in the hepatocyte, pancreatic β cells, podocytes, and cardiomyocytes [15–17, 30] revealed its lysosomal phenotypes. The regulative relationship between ATP6AP2, autophagic flux, and NLRP3 under pressure load is poorly understood. There is still much debate about the ATP6AP2 function, and more research is needed.

Consequently, we hypothesized ATP6AP2 could exert its protective role by promoting autophagic flux and further inhibiting NLRP3 activation. Our results firstly demonstrated that mice with ATP6AP2 knockdown in the heart compromise autophagic flux, activate NLRP3, and further promote maladaptive cardiac remodeling in the TAC model. Cellular ROS and mitochondrial ROS induced by ATP6AP2 knockdown accelerate NLRP3 activation.

## Results

### Dynamic changes of ATP6AP2 in the progression toward heart failure

**Fig. 1:**
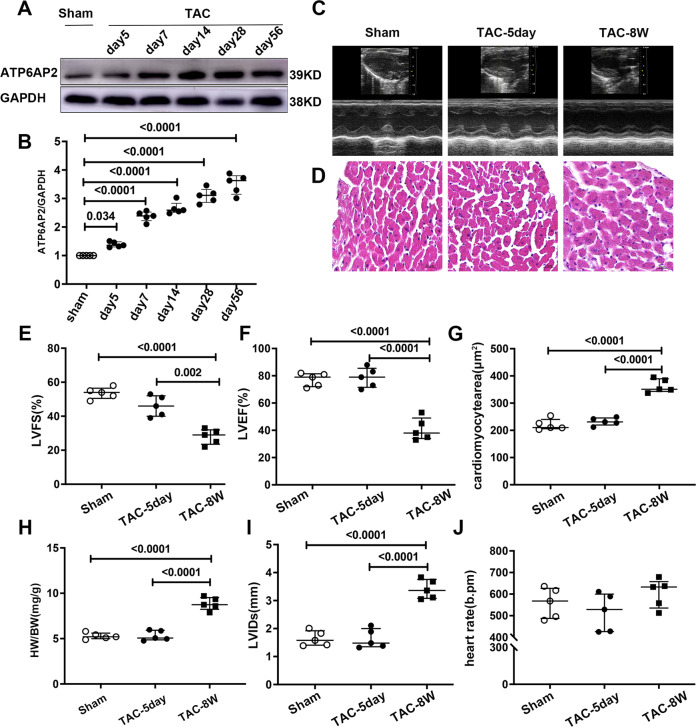
Dynamic changes of ATP6AP2 in the progression toward heart failure. **A**, **B** Representative immunoblots of ATP6AP2 expression in the heart tissues from sham (*n* = 5) or TAC groups (*n* = 5 for each group) and quantitative analysis are shown. Statistical analysis was conducted by Kruskal–Wallis one-way ANOVA with Dunn post-hoc test. **C** Representative images of echocardiograms of sham and TAC mice at different timepoints. **D** Representative hematoxylin and eosin staining of cardiomyocyte staining (scale bar = 20 μm). **E**, **F**, **I**, **J** quantitative analysis of heart function of M-mode echocardiograms and heart rate. **G** Quantitative analysis of cardiomyocytes cross-sectional area. **H** Quantitative analysis of heart weight/body weight ratios (HW/BW; mg/g). Statistical analysis was conducted by Kruskal–Wallis one-way ANOVA with Dunn post-hoc test. Data were presented as medians and quartiles.

To confirm the changed expression of ATP6AP2 during the hypertrophy and heart failure phase, wild-type C57BL/6 J mice were subjected to transverse aortic constriction (TAC) [18]. Measurements of peak flow velocity through the constricted aortic arch were similar on the 5th and 56th day past-TAC, which indicated the surgery was successful (Fig. S2ACDE). The echocardiography showed that LV ejection fraction (LVEF), LV fraction shortening (LVFS), LV internal diameter at end-systole (LVIDs) did not change significantly on the 5th day. (Fig. 1C, E, F, I). By the 56th day, LVEF and LVFS dropped by 35–40% (Fig. 1E, F). Meanwhile, LVIDs, HW/BW, and cardiomyocyte cross-sectional area increased considerably (Fig. 1D, G, H, I). Heart rate remained similar between groups (Fig. 1J).

**Fig. 2:**
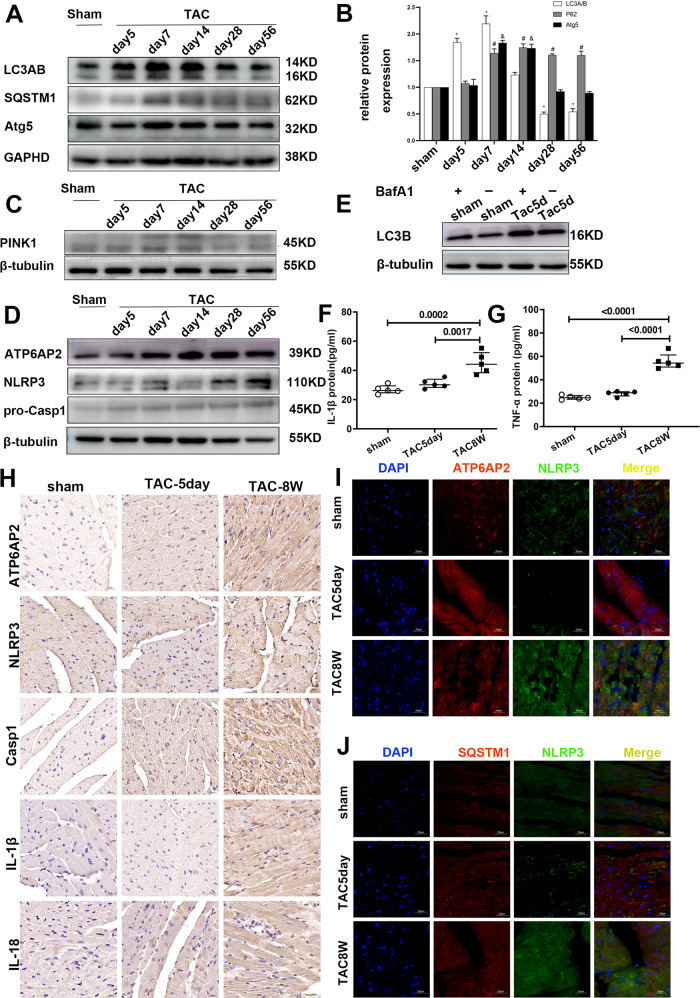
General autophagy and mitophagy are transiently upregulated in hypertrophic hearts but are decreased during decompensated heart failure combined with NLRP3 inflammasome upregulation. Representative immunoblots and quantitative analysis of LC3A/B and P62/SQSTM1, Atg5 (**A**, **B**), PINK1 (**C**), ATP6AP2, NLRP3, pro-caspase1 (**D**) are shown from whole-cell heart homogenates (*n* = 5 for each group). Statistical analysis was conducted by Kruskal–Wallis one-way ANOVA with Dunn post-hoc test. For LC3AB: *P < 0.05 vs. sham-operated mice. For P62/SQSTM1: ^#^*P* < 0.05 vs. sham-operated mice. For Atg5: ^&^*P* < 0.05 vs. sham-operated mice. At least four independent experiments were conducted. **E** Representative immunoblots for autophagic flux measure of whole-cell heart homogenates for LC3AB with or without BafA1. **F**, **G** Quantitative analysis of ELISA measurement of serum levels of IL-1β, TNF-α expression levels. Statistical analysis was conducted by Kruskal–Wallis one-way ANOVA with Dunn post-hoc test. Data were presented as medians and quartiles. **H** Representative immunohistochemical staining of ATP6AP2, NLRP3, Caspase1, IL-1β, IL-18 in sham groups, TAC5day, TAC56day.(scale bar = 20 μm). **I**, **J** Representative immunofluorescence images of colocalization of NLRP3 with ATP6AP2 or SQSTM1/P2, with NLRP3 in tissue section.

ATP6AP2 expression began to increase on the 5th day of TAC and increased sharply from the 7th day. After that, the expression remained at a higher level (Fig. 1A, B). Immunohistochemical staining (IHC)of ATP6AP2 also confirmed this trend (Fig. 2H).

### General autophagy and mitophagy are transiently upregulated in the hypertrophic heart but are decreased during the decompensated heart failure

Based on LC3, P62 (SQSTM1), Atg5, we first detected the changes in general autophagy at different time points after TAC. LC3B began to upregulate on the 5th and the 7th but returned to the basal level on the 14th day. However, it declined significantly after 28 days. SQSTM1/P62 increased from 7 days and was maintained at high levels (Fig. 2A, B).

The activation of autophagy flux or blockage of the degradation of autolysosome both can increase the expression of steady-state LC3B. To distinguish between the possibilities, we treated mice with Bafa1 intraperitoneally, a late-stage autophagy inhibitor. After BafA1 treatment, the level of LC3B increased considerably 5 days after TAC compared with the TAC group (Fig. 2E). These results indicated autophagic flux was activated on the 5th day of TAC when the heart was still in compensated phase. PINK1(PTEN-induced putative kinase 1) generally indicates the degree of mitophagy. Similar to previous research [19], our results showed cytosol PINK1 downregulated considerably after TAC 28 days (Fig. 2C).

### NLRP3 expression elevates combined with blocked autophagic flux in decompensatory heart failure

Next, we want to explore the relationship between ATP6AP2, NLRP3 inflammasome, and autophagic flux during the TAC-induced early and late failure stages. Western blotting and IHC showed expression of NLRP3, caspase1, IL-1β, IL-18 increased in the myocardial tissue of TAC8w mice (Fig. 2D, H). ELISA results confirmed that serum IL-1β and TNF-α concentrations were significantly upregulated (Fig. 2F, G). Tissue fluorescence double staining showed that the colocalization of NLRP3 and ATP6AP2 increased (Fig. 2I), also the colocalization of NLRP3 and SQSTM1/P62 increased in TAC8W myocardium (Fig. 2J). These changes did not happen on TAC 5 days except for some increment of ATP6AP2 expression. Overall, these changes indicated that during decompensated heart failure, NLRP3 activated, accompanied by blockage of autophagic flux.

### Deletion of ATP6AP2 exacerbates progression of TAC-induced hypertrophy to heart failure in mice accompanying NLRP3 inflammasome expression upregulation

**Fig. 3:**
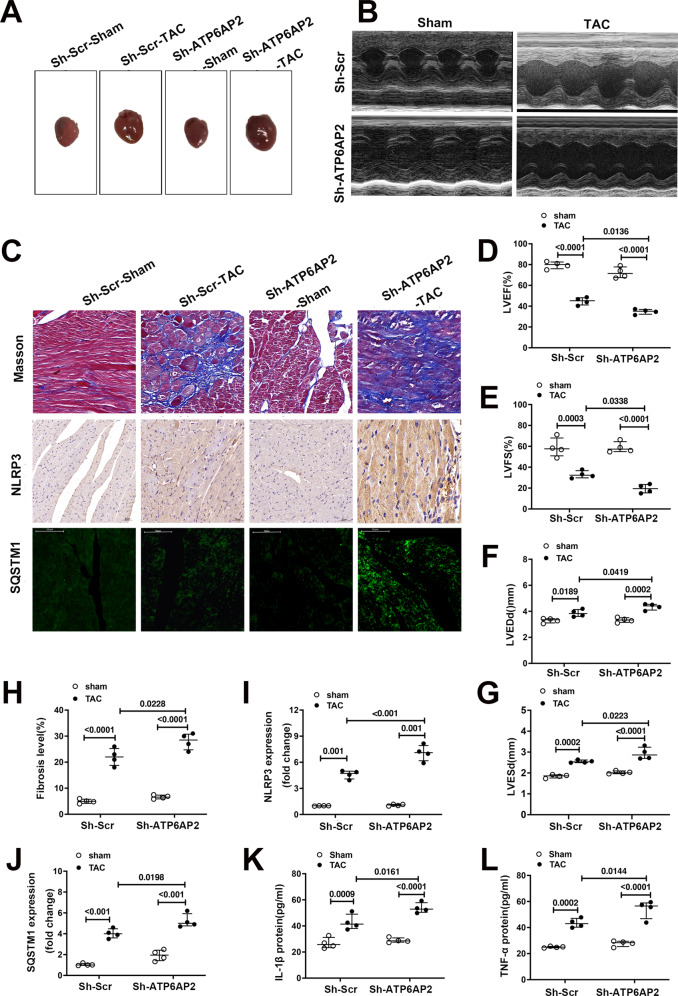
Deletion of ATP6AP2 exacerbates progression of TAC-induced hypertrophy to heart failure in mice accompanying NLRP3 inflammasome expression upregulation. **A** The gross morphology of mice hearts in different groups is shown. **B**, **D**, **E**, **F**, **G** Representative images of echocardiograms and quantitative analysis of LVEF, LVFS, LVEDD, and LVESD between different groups (*n* = 4 for each group). Statistical analysis was conducted by Kruskal–Wallis one-way ANOVA with Dunn post-hoc test. **C**, **H** Representative images of Masson trichrome staining of mice hearts and quantitative analysis. Statistical analysis was conducted by Kruskal–Wallis one-way ANOVA with Dunn post-hoc test. **C**, **I**, **J** Representative immunohistochemical staining and quantitative analysis about staining of NLRP3 and immunofluorescence images of SQSTM1/P62. Statistical analysis was conducted by Kruskal–Wallis one-way ANOVA with Dunn post-hoc test. **K**, **L** quantitative analysis of ELISA measurement of serum levels of IL-1β, TNF-α expression levels. Statistical analysis was conducted by Kruskal–Wallis one-way ANOVA with Dunn post-hoc test. Data were presented as medians and quartiles.

To confirm whether the increased expression of ATP6AP2 exerts pathological effects or the upregulation is just a compensated increment with heart failure, and adenovirus-mediated vector for ATP6ap2 knockdown(shRNA-ATP6AP2) or control (shRNA-Src) was constructed. Western blot showed about 75–80% knockout efficiency (Fig. S3AB). Basal cardiac function showed no differences between mice receiving shRNA-ATP6AP2 or shRNA-Src. Four weeks after TAC, cardiac dysfunction was severe in shRNA-ATP6AP2-TAC.This effect was reflected by increased heart weight (Fig. 3A, Table. S3) and HW/BW, decreased LVEF and LVFS, and increased LVEDD, LVESD (Fig. 3B, D, E, F, G). Also, Masson’s trichrome showed prominent fibrosis in sh-ATP6AP2-TAC (Fig. 3C, H). IHC staining showed increased staining for NLRP3 in the shR-ATP6AP2-TAC group. (Fig. 3C, I). Meanwhile, tissue immunofluorescence(IF) indicated increased SQSTM1/P62 in the sh-ATP6AP2-TAC group (Fig. 3C, J). Serum IL-1β and TNF-α showed apparent upregulation (Fig. 3K, L). The results indicated that the upregulation of ATP6AP2 under pressure overload might be a compensatory response.

### Moderately improved autophagic flux but similar fibrosis and cardiac dysfunction in Tg-ATP6AP2-TAC mice

**Fig. 4:**
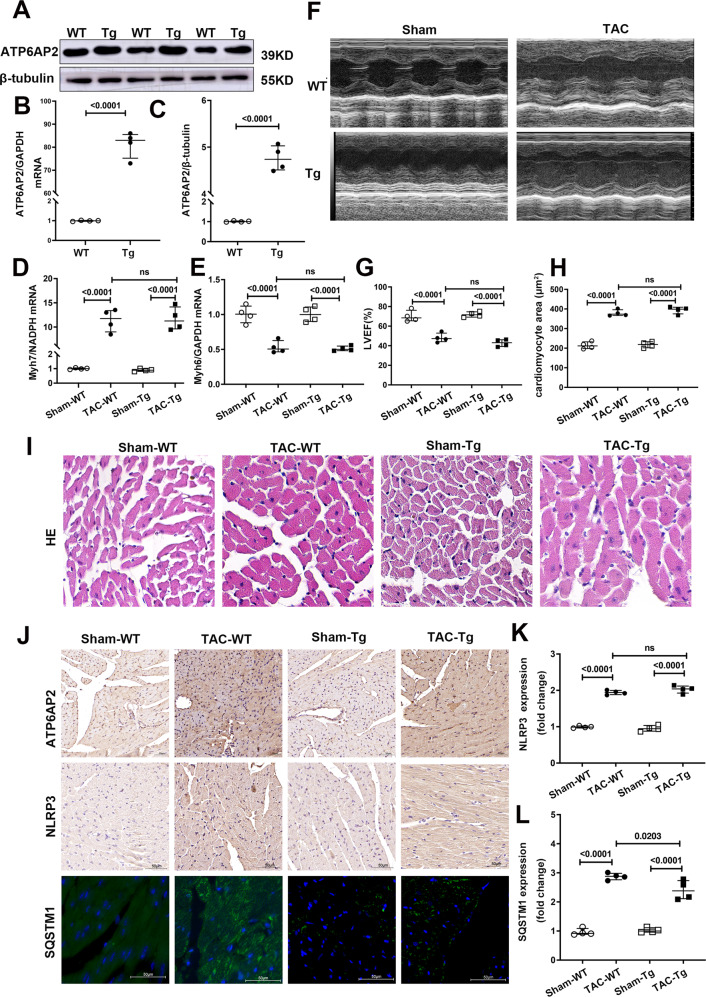
Moderately improved autophagic flux but similar fibrosis and cardiac dysfunction in Tg-ATP6AP2 mice. **A–C** Representative immunoblots and quantitative analysis of whole-cell heart homogenates for ATP6AP2. (*n* = 4 for each group). Statistical analysis was conducted by the Mann–Whitney test. Quantitative analysis Myh6, Myh7 (**D**, **E**) expression of whole-heart homogenates by qRT-PCR. Statistical analysis was conducted by Kruskal–Wallis one-way ANOVA with Dunn post-hoc test. **F**, **G** Representative images of echocardiograms and quantitative analysis of LVEF between different groups. Statistical analysis was conducted by Kruskal–Wallis one-way ANOVA with Dunn post-hoc test. **H**, **I** Representative hematoxylin and eosin staining and quantitative analysis of cardiomyocytes cross-sectional area (scale bar = 20 μm). Statistical analysis was conducted by Kruskal–Wallis one-way ANOVA with Dunn post-hoc test. **J**, **K**, **L** Representative immunohistochemical staining and quantitative analysis about staining of ATP6AP2, NLRP3 and immunofluorescence images of SQSTM1/P62. Statistical analysis was conducted by Kruskal–Wallis one-way ANOVA with Dunn post-hoc test. Data were presented as medians and quartiles.

To further confirm the effect of ATP6AP2 upregulation in heart failure, we generated ATP6AP2 gain-of-function transgenic mice (Fig. S1). mRNA expression increased more than 80 folds, and protein expression increased about five folds (Fig. 4A-C) in the hearts of Tg-ATP6AP2 mice compared with wild littermates. There were no baseline differences in heart weight/body weight ratio (HW/BW; mg/g), the cardiac function between the two groups (Table S2).

After TAC was performed, the mRNA levels of Myh6, Myh7 did not show a difference between TAC-Tg and TAC-WT (Fig. 4D, E). Echocardiography showed LVEF were identical (Fig. 4F, G). Myocyte’s surface area was determined by HE staining; no difference was observed between TAC-Tg and TAC-WT (Fig. 4H, I). Also no significant difference in IHC staining for NLRP3, Caspase1, IL-1β, IL-18 (Fig. 4J, K, Fig. S2BFGH). However, IHC/IF indicated downregulation of SQSTM1/P62 expression in TAC-Tg relative to TAC-WT (Fig. 4J, L). This suggested that ATP6AP2 overexpression reduced the inhibition of autophagic flux. The results indicated that the upregulation of ATP6AP2 may be compensated response to alleviate the blocked autophagic flux.

### shR-ATP6AP2 increases cell death partly through activation of NLRP3 inflammasome in vitro

We have proved that ATP6AP2 knockdown increased NLRP3 expression in TAC-induced heart failure. In vitro research was performed to explore the mechanism. We firstly examined the ATP6AP2 expression in phenylephrine-induced primary isolated neonatal rat ventricular myocytes (NRVMs). NRVMs were stimulated with phenylephrine (100 μM) at different timepoints to mimic alterations in failing hearts in vivo. Phenylephrine stimulation for 6 h induced the upregulation of ATP6AP2, then continued to rise and maintained a high level throughout 48 h (Fig. S3C). Meanwhile, SQSTM1/P62 or NLRP3 did not show significant change when 6 h of phenylephrine but a marked upregulation after 48 h. Hence we selected phenylephrine stimulation for 6 h to mimic the early hypertrophy stage and 48 h to mimic the late-phase failure, respectively.

**Fig. 5:**
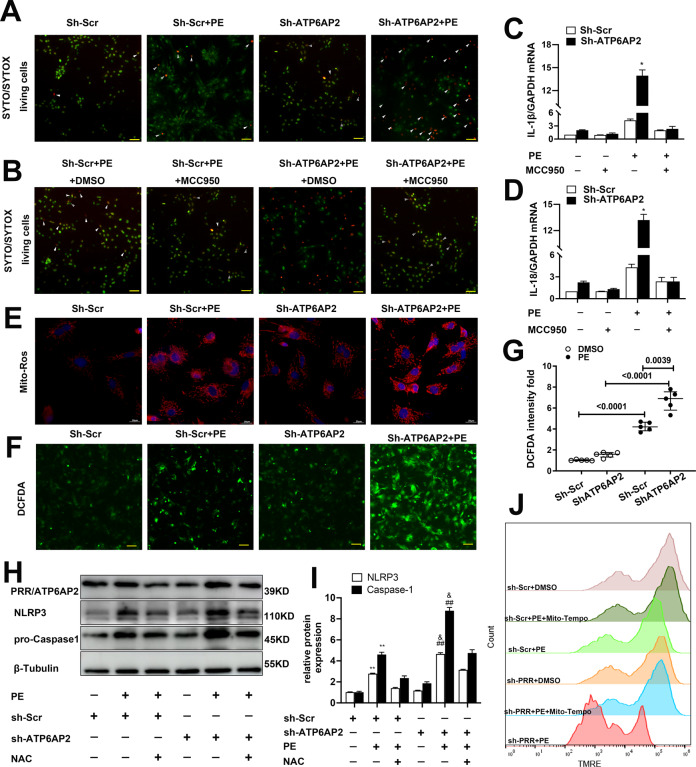
Oxidative stress and mitochondrial ROS promote NLRP3 inflammasome activation and further increase cell death in shR-ATP6AP2-transfected NRVMs. **A**, **B** Representative images of NRVMs stained with SYTOX ^TM^ Orange or SYTO^TM^ Green. Green dots indicated live cells, and orange dots indicated dead cells (white arrowheads). NRVMs transfected shR-ATP6AP2 showed more phenylephrine-induced cell death. While NRVMs pretreated with MCC950 showed alleviated cell death (scale bar = 50 μm). **C**, **D** indicates quantitative analysis IL-1β and IL-18 using quantitative real-time PCR. Statistical analysis was conducted by one-way ANOVA with Tukey post-hoc test. At least four independent experiments were conducted. **E** Representative fluorescent images of NRVMs mitochondrial ROS production measured by MitoSOX. The shR-ATP6AP2 + phenylephrine group showed increased mitROS compared with the shR-Scr+phenylephrine group. **F**, **G** Representative fluorescent images of NRVMs total ROS production by DCFDA and quantitative analysis. Statistical analysis was conducted by Kruskal–Wallis one-way ANOVA with Dunn post-hoc test. Data were presented as medians and quartiles. **H**, **I** Representative immunoblots and quantitative analysis of lysates of NRVMs between groups indicated NAC inhibited shR-ATP6AP2 + phenylephrine-induced NLRP3 upregulation. **P < 0.01 vs. shR-Scr group, ^##^*P* < 0.01 vs. shR-ATP6AP2, ^&^*P* < 0.05 vs sh-ATP6AP2 + phenylephrine+NAC. Statistical analysis was conducted by one-way ANOVA with Tukey post-hoc test. At least four independent experiments were conducted. **J** Representative images of TMRE by Flow cytometry indicated that mitochondrial membrane potential was decreased by shR-ATP6AP2 under phenylephrine stimulation while partly rescued by Mito-Tempo.

Long-time phenylephrine stimulation can induce cellular death (Fig. 5A, orange dots or white arrowheads indicated). This is similar to the previous study [7]. However, NRVMs transfected shR-ATP6AP2 showed more susceptibility than sh-Scr NRVMs to phenylephrine-induced cell death (Fig. 5A). This tendency was rescued after NLRP3 inhibitor MCC950 was given (Fig. 5B). Also, mRNA expression of IL-1βand IL-18 decreased (Fig. 5C, D). Next, we tend to further explore what mechanism ShR-ATP6AP2 causes upregulation of NLRP3.

### Oxidative stress and mitochondrial ROS were required for NLRP3 inflammasome activation in ATP6AP2 knockdown cardiomyocytes

As ROS is recognized as one of the most common mechanisms in NLRP3 activation, we further detected whether oxidative stress involves shR-ATP6AP2 induced pathological phenotype. MitoSOX and DCFDA were used to measure mitochondrial ROS and total ROS in 48 h of phenylephrine stimulated NRVMs (Fig. 5E, F). Phenylephrine-induced upregulation of mito-ROS and total ROS compared with DMSO control. However, more ROS was produced in the shR-ATP6AP2 + phenylephrine group (Fig. 5E–G). After blocking ROS with N-acetylcysteine (NAC), NLRP3 and Caspase1 downregulated considerably compared with phenylephrine+sh-ATP6AP2 (Fig. 5H, I). Also, TMRE by flow cytometry indicated mitochondrial membrane potential impaired by phenylephrine; shR-ATP6AP2/PRR aggravated this impairment. However, this change was rescued after blocking mitochondrial ROS with Mito-Tempo (Fig. 5J).

### ATP6AP2 is required for the completion of autophagy and its knockdown inhibited V-ATPase-Driven lysosome acidification

**Fig. 6:**
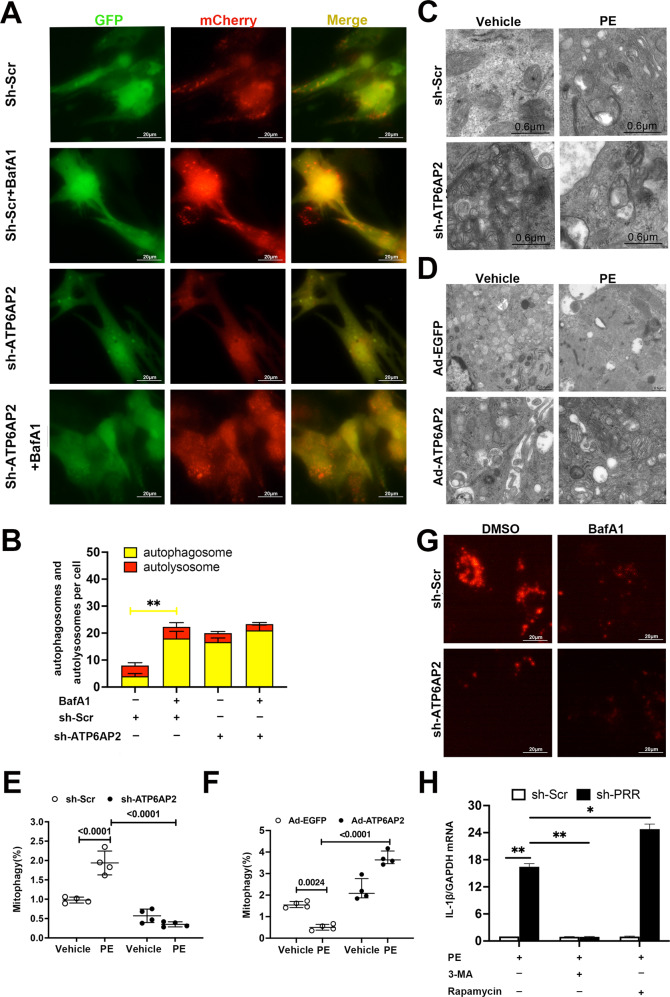
ATP6AP2 is required for the completion of autophagy and its knockdown inhibits V-ATPase-driven lysosome acidification. **A**, **B** Representative fluorescent images are shown. Manually quantitative analysis indicates the mean number of autophagosomes (yellow) and autolysosomes (red) per cell. Statistical analysis was conducted by Kruskal–Wallis one-way ANOVA with Dunn post-hoc test. **P < 0.01. **C**, **D** Autophagy and mitophagy levels showed by transmission electron microscopy in NRVMs transfected with shR-ATP6AP2 knockdown vector or Ad-ATP6AP2 overexpression vector. Representative images of normal mitochondria and mitochondria in-taken by vacuole structure can be seen. **E**, **F** indicated mitophagy percentage reflected the number of autophagosomes containing mitochondria per total number of mitochondria from a cross-sectional assessment of the NRCMs. Statistical analysis was conducted by Kruskal–Wallis one-way ANOVA with Dunn post-hoc test. Data were presented as medians and quartiles. **G** Representative fluorescent images of LysoTracker Red staining in shR-Scr and shR-ATP6AP2 NRVMs treated with DMSO or bafilomycin A1 for 60 min. **H** the effect of autophagy inducer or inhibitor on IL-1β mRNA expression in NRVMs while sh-ATP6AP2 was transfected. GAPDH was used as the loading control. Statistical analysis was conducted by Kruskal–Wallis one-way ANOVA with Dunn post-hoc test. At least four independent experiments were conducted. *P < 0.05, **P < 0.01.

Next, we tended to reveal cellular autophagy changes of shR-ATP6AP2 transfected NRVMs. We transduced an adenovirus plasmid encoding mCherry-GFP-LC3 into sh-Scr and sh-ATP6AP2 NRVMs. Using this reporter, we examined autophagic flux. BafA1, which inhibits autophagosome-lysosome fusion [19] was used. BafA1 triggered a significant increase of autophagosomes indicated by yellow spots in the ShR-Scr group. In shR-ATP6AP2 NRVMs, more autophagosomes can be seen on the baseline. However, the increment of autophagosomes indicated by the yellow spots percentage after BafA1 in shR-ATP6AP2 was comparable to shR-ATP6AP2 (Fig. 6A, B). This indicated that shR-ATP6AP2 affected late autophagic flux.

Transmission electron microscopy (TEM) showed that the percentage of mitophagy in NRVMs increased after 6 h of phenylephrine stimulation. There was a significant accumulation of multivesicular vacuoles (Fig. 6C). Swollen or partially digested mitochondria can be seen around nuclear in large autophagic vacuoles. However, more undigested mitochondria can be seen in shR-ATP6AP2 + PE (Fig. 6C, E). Meanwhile, 48 h of phenylephrine inhibited mitophagy in NRVMs (Fig. 6D, F). However, the percentage of mitophagy was increased after the Ad-ATP6AP2 overexpression vector was transfected. Compared with the Ad-EGFP + phenylephrine group, more mitochondria were incorporated into autophagic vacuoles and digested in the PE + Ad-ATP6AP2 group.

Lysotracker Red probe (100 nmol/L for 30 min) showed that positive puncta in the cytosol were significantly reduced in the sh-ATP6AP2 group compared with shR-Src. Bafilomycin A1 (BafA1) treatment (100 nmol/L for 4 h) served as a positive control (Fig. 6G). Treated sh-ATP6AP2 NRVMs with 3-methyladenine (an autophagy inhibitor) or rapamycin (an autophagy inducer), inhibited or enhanced the expression of the cytokine mRNA of IL-1β in NRVMs treated with 6 h of phenylephrine, respectively (Fig. 6H). These results also confirm ATP6AP2 exerts its role at the late stage of autophagic flux.

### Phenotype changes in sh-ATP6AP2-TAC can be partly relieved by blocking NLRP3 inflammasome activation

**Fig. 7:**
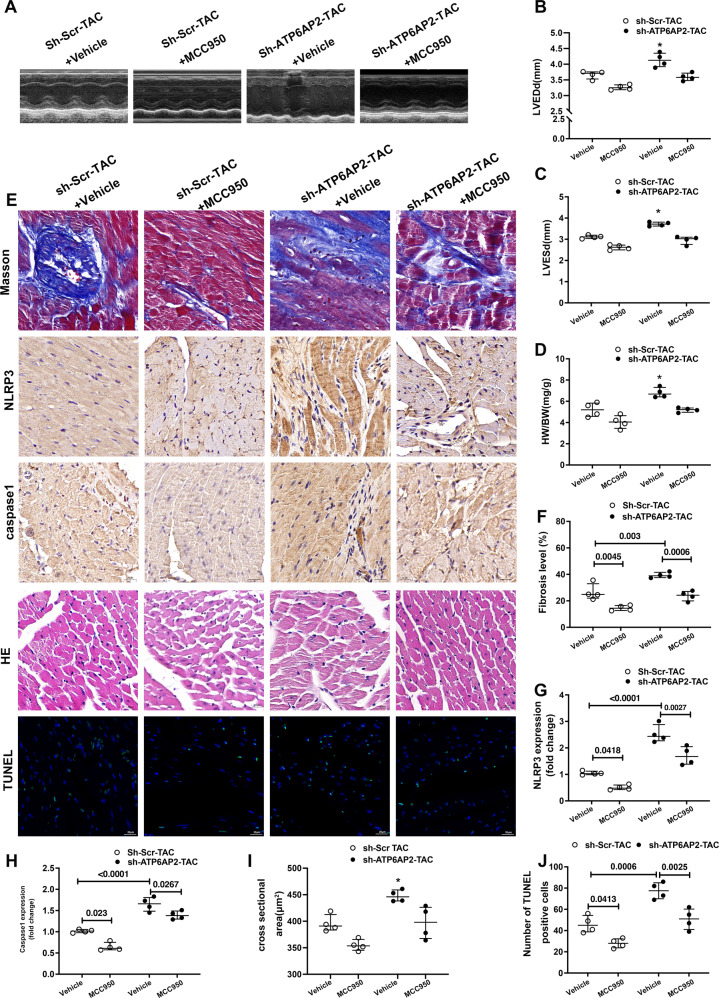
Phenotype changes in shR-ATP6AP2-TAC mice can be partly relieved through blocking NLRP3 inflammasome activation. C57BL/6 J mice were subjected to TAC, then adenovirus-mediated shR-ATP6AP2 vectors or Scr-control were injected into the tail vein of mice (*n* = 4 for each group), followed by MCC950 (5 mg/kg/day) or vehicle control. Mice were observed at 28days. **A**–**C** Representative images of echocardiograms and quantitative analysis of LVEDD, LVESD between different groups. Statistical analysis was conducted by Kruskal–Wallis one-way ANOVA with Dunn post-hoc test. *P < 0.05 compared with others. **D** Quantitative analysis of heart weight/body weight ratios (HW/BW; mg/g). Statistical analysis was conducted by Kruskal–Wallis one-way ANOVA with Dunn post-hoc test. *P < 0.05 compared to other groups. **E**, **F** Representative images of Masson trichrome staining and quantitative analysis. Statistical analysis was conducted by Kruskal–Wallis one-way ANOVA with Dunn post-hoc test. **E**, **G**, **H** Representative immunohistochemical staining and quantitative analysis of NLRP3, Caspase1 expression. (scale bar = 20 μm). Statistical analysis was conducted by Kruskal–Wallis one-way ANOVA with Dunn post-hoc test. **E**, **I** Representative hematoxylin and eosin (HE) staining and quantitative analysis of cardiomyocytes cross-sectional area (scale bar = 20 μm). Statistical analysis was conducted by Kruskal–Wallis one-way ANOVA with Dunn post-hoc test. *P < 0.05 compared with other groups. **E**, **J** Representative immunofluorescence images of TUNEL assay showed apoptosis percentage in mouse heart tissues. Statistical analysis was conducted by Kruskal–Wallis one-way ANOVA with Dunn post-hoc test. Data were presented as medians and quartiles.

We next examined whether the inhibition of NLRP3 can rescue the pathological cardiac phenotypes in TAC-operated sh-ATP6AP2 mice. MCC950 attenuated chamber dilation and cardiac dysfunction revealed decreased LVEDD, LVESD compared with the sh-ATP6AP2-TAC 28 days after TAC (Fig. 7A–C). Also, HW/BW decreased after MCC950 was administrated (Fig. 7D). Masson’s trichrome indicated that administration of MCC950 resulted in the decrease of fibrosis at 28 days after TAC (Fig. 7E, F). We can see NLRP3 and casapse1expression were significantly reduced after MCC950 was given compared with the sh-ATP6AP2-TAC group (Fig. 7E, G, H). HE staining and TUNEL also indicated MCC950 caused a reduction of cardiomyocyte area and apoptosis after MCC950 was given (Fig. 7E, I, J).

## Discussion

**Fig. 8:**
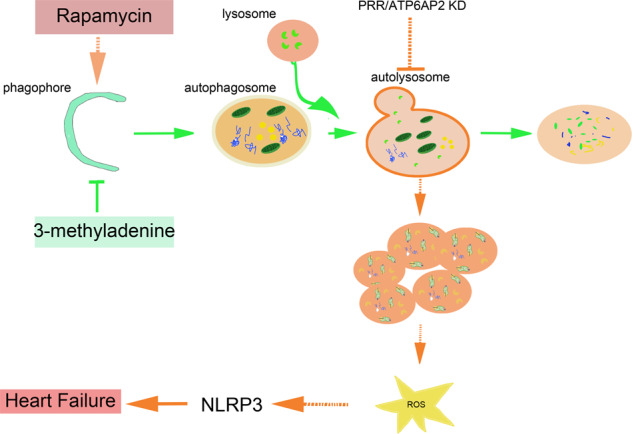
Working model. ATP6AP2, maintaining lysosomal acidification function. When ATP6AP2 was knockdown, cardiomyocyte autophagic flux and mitophagy were blocked. Further leads to the accumulation of dysfunctional mitochondria, increasing reactive oxygen species (ROS), or mitochondrial ROS. Meanwhile, accumulated ROS activates the NLRP3 inflammasome and leads to heart failure. Promoting autophagosome formation via Rapamycin increase NLRP3 activation in shR-ATP6AP2-transfected cardiomyocytes. Conversely, slowing autophagy initiation by 3-MA partially reduces ROS and activation of NLRP3 in shR-ATP6AP2-transfected cardiomyocytes. Green solid arrows show the normal autophagic flux. Arrows in dotted orange indicate that knockdown of ATP6AP2 gene blocks autophagic flux, accumulated undigested mitochondria trigger ROS and NLRP3.

In the present study, we investigate the regulative relation of ATP6AP2, autophagic flux, and NLRP3 inflammasome activation in the progression of cardiac hypertrophy and heart failure. We obtain the following principal findings. (i) Knockdown of ATP6AP2 deteriorates heart function in TAC-induced heart failure. (ii) Knockdown of ATP6AP2 compromises lysosomal acidification, hence impaired autophagic flux and mitophagy. (iii) NLRP3 inflammasome significantly increases in TAC-induced heart failure after ATP6AP2 knockdown. The NLRP3 inflammasome partially induces myocardial fibrosis and cardiac dysfunction. NLRP3 inhibition has a definite protective effect in ATP6AP2 knockdown-induced heart failure. (iv) Blocking cellular ROS and mitochondrial ROS of shR-ATP6AP2-transfected cardiomyocytes, can partially rescue the upregulation of NLRP3 and mitochondrial damage (Fig. 8).

The (pro)renin receptor ((P)RR), gene name ATP6AP2, was initially recognized as a potential regulator role in the renin-angiotensin system (RAS). It is ubiquitously expressed in the kidney, heart, brain and immune system, and so on [31]. It participates in a series of physiological processes, such as the cell cycle, autophagy, energy metabolism, and exerts a pathological role in fibrosis, hypertension, insulin resistance, pre-eclampsia, kidney injury, and cardiovascular diseases [32]. Nevertheless, there remains controversy about its function in pathological processes. Mice with cardiac-restricted overexpression of ATP6AP2 failed to produce morphological abnormalities under basal state and isoproterenol infusion [28, 29]. In physiological situations such as pregnancy, although high ATP6AP2 maternal plasma level, apparent tissue damage was absent [33]. Some research implements loss of function on ATP6AP2 through handle region peptide (HRP). However, there remains controversy about HRP efficiency [34, 35].

Despite two decades of studies on ATP6AP2, its function has yet to be fully revealed [20]. So far, there is some research about the role of ATP6AP2 in modulating lysosomal acidification as a component of V-ATPase. Most targeted tissues, cell homeostasis was compromised [15, 17, 30, 36, 37]. When ATP6AP2 knockout was specifically performed in cardiomyocytes or podocytes, mice developed lethal heart failure, nephrotic syndrome, and albuminuria. The most obvious changes in the cell are accumulated autophagic vacuoles and cytoskeletal changes [16, 30]. In humans, ATP6AP2 mutations failed to recognize any evidence of its role in the RAS [38]. Instead, links between ATP6AP2 missense mutations with autophagic defeats emerged significantly [39, 40].

According to previous articles, in our study, autophagic flux was measured as objectively as possible [41–43]. Several combined methods were selected for a comprehensive evaluation. Our results show ATP6AP2 upregulation can be seen from the hypertrophic phase until heart failure. ATP6AP2 knockdown blocked autophagic flux at late-phase autolysosome by compromising V-ATPase. This is consistent with the previous study [44]. We speculate this compensatory ATP6AP2 upregulation may exert its physiological pro-autophagic and protective role in pressure overload stress. The clinical application of recombinant BNP in severe decompensated heart failure inspired us. Even though serum BNP is at a high level, it still does not meet the compensatory needs in this situation. The exogenous input can effectively improve symptoms because the concentration of endogenous BNP and exogenous input is not in the same order of magnitude.

Mitochondrial impairment and dysfunction can be provoked by prolonged and high-level cardiac stress. As one of the three most important processes for mitochondrial quality control, Mitophagy is adaptively induced to remove the damaged mitochondria and prevent oxidative damage [45]. Autophagy and mitophagy can inhibit apoptosis and postpone progression to heart failure to some extent [46, 47]. B. Wang et al. showed that enhancing mitophagy by the AMPKα2-PINK1-PARKIN pathway improved TAC-induced heart failure [19]. Our results revealed that ATP6AP2 knockdown or overexpression accordingly obstructed or facilitated autophagy flux. This result is consistent with previous research [15, 17, 30]. We further revealed that mitophagy is also mediated by ATP6AP2.

NLRP3 inflammasome pathways are closely related to most cardiovascular diseases. Cardiac fibrosis and multiple sclerosis severity can be limited by NLRP3 inflammasome inhibitor [48]. ASC^−/−^ mice had decreased neointimal lesion, the neointimal formation was attenuated after vascular injury [49]. The acute or chronic inflammation aroused by cholesterol crystals or a high-cholesterol diet was limited in NLRP3 or IL-1β-deficient mice [12, 13, 50, 51]. Pressure overload, especially in the early phase, can trigger CaMKIIδ-NLRP3 pathway activation in cardiomyocytes and recruit macrophages to promote fibrosis and cardiac dysfunction [14]. Mitochondrial DNA, if not be eliminated appropriately and timely by autophagy, can trigger IL-1β mediated myocarditis and dilated cardiomyopathy via TLR9 activation [8]. Increasing evidence indicates the crosstalk between autophagy and inflammasome. Autophagy can downregulate NLRP3 inflammasome activation and exert protective effects [52–54]. Consistent with these researches, our results affirmed that NLRP3 promotes cardiac fibrosis and dysfunction in pressure overload stress. We further revealed that ATP6AP2 could promote autophagic flux and anti-inflammasome function.

Oxidative stress has been widely accepted to contribute to cardiac remodeling in many pathological processes such as cardiac hypertrophy, cell apoptosis [55]. Defective autolysosomes, damaged mitochondria that not be timely degraded can be the source of ROS [56]. Excessive ROS can provoke doxorubicin cardiotoxicity, myocardial infarction, ischemia-reperfusion injury [12, 51, 57–60]. The high efficiency of autophagic flux may play a protective mechanism against excessive ROS in doxorubicin-induced cardiomyopathy [6, 61, 62]. Our in vitro results indicated that ATP6AP2 knockdown induced autophagic dysfunction and dysfunctional mitochondria accumulation, increased ROS and mitochondrial ROS further activated NLRP3 inflammasome. Our results are consistent with previous studies. Again we confirm autophagy has a suppressive role on NLRP3 by inhibiting oxidative stress.

In consideration of cardiac diseases diversity and severity, and there still exist some inconsistent implementation of assays to assess autophagy in different contexts, some variability was revealed in changed autophagic activity among different animal models. We tried to perform longitudinal experiments at different time points of the TAC model to sketch altered autophagic activity, including early hypertrophy and late heart failure stages. As ATP6AP2 and autophagy may play multifaceted and complex roles in disease pathogenesis, much work still needs to be done.

In conclusion, our research shows that mice with ATP6AP2 knockdown in cardiomyocytes compromising autophagic flux confers a more obvious inflammasome activation phenotype and further promote maladaptive cardiac remodeling in the model of pressure overload stress. Cellular ROS and mitochondrial ROS induced by ATP6AP2 knockdown accelerate NLRP3 activation. The clinical research CANTOS involving IL-1β neutralizing antibody canakinumab in coronary disease has encouraging findings. It predominantly decreased myocardial infarction [63]. This may give us some inspiration that NLRP3 inflammasome inhibition can be particularly effective to prevent this maladaptive remodeling in situations where the autophagic flux is not fully compensated in late heart failure. Cell impairment triggered by ATP6AP2 depletion argues against using ATP6AP2/PRR inhibitors for treatment.

## Methods

### Neonatal rat ventricular myocyte isolation (NRVMs)

Hearts from 1–3-day-old rat pups of Sprague-Dawley were isolated and digested using collagenase II (C8150, Solarbio). The cell suspension was preplated in the culture flask and adhered to for 1.5 h at differential speed. Through this procedure, purified myocytes can be obtained. Then plated on gelatin-coated plates at a density of 4 × 10^4^cell/cm^2^ overnight at 37 °C. the final concentration of 100 μmol/L Bromodeoxyuridine was added to the plating medium containing 10% FBS. Cardiomyocyte purity was about 95%. NRVMs were continued to culture in a complete medium for 3days (high-glucose DMEM containing 5% FBS,100 units/ml penicillin, and 100 μg/ml streptomycin), experiments were initiated on the fourth day.

Adenovirus expressing the constitutively active ATP6AP2(Ad-ATP6AP2) or EGFP(Ad-EGFP) and Sh-ATP6AP2, Sh-Scr was applied to the culture at a multiplicity of infection (MOI) of 50 for 24 h.

### Drug treatment

Phenylephrine (PE 100μmol/L) was added to the Cardiomyocytes medium at a series of time points, followed by carbonyl cyanide p-trichloromethoxyphenyl hydrazone CCCP (20 μM) treatment to induce mitophagy. Some may be pretreated with or without a small molecular inhibitor of NLRP3-MCC950(5 uM) [12], antioxidant N-acetylcysteine NAC (10 mM), Mito-Tempo (500 uM). In some experiments, mice were injected MCC950 (5 mg/kg/day) diluted in PBS via intraperitoneal injection [64] or Vehicle control (PBS).

### Recombinant adenovirus vectors

Recombinant adenovirus vectors-mediated ATP6AP2 knockdown was designed by Gene-Pharma (Shanghai Gene Pharma Co. Ltd, Shanghai, China). We synthesized interfering sequences targeting ATP6AP2:5’- GCTCCGTAATCGCCTGTTTCA-3’.Adenovirus containing rat ATP6AP2 cDNA(gene ID: 302526) sequence or control transgene Ad-EGFP was synthesized and cloned into pDC315 with the EcoR I and Sal I sites as previously described using the AdMax system [65]. Adenovirus is administered through the tail vein in 10^9^ doses per mouse. The expression time of the adenovirus vector in vivo was about 2–4 weeks, and we gave the same dose of virus again 2 weeks later.

### Immunoblot analysis

Forty milligrams Ventricular tissue was homogenized in 300 ul protein lysis buffers (P0013B, beyotime). Protease inhibitors (PMSF36978, Thermo Scientific^TM^) and Phosphatase inhibitors were added before use. Centrifugation was performed at 10,000–14,000 × *g* for 3–5 min after full lysis. The supernatant was taken for subsequent Western.

For NRVMs, remove the culture medium and wash with cold PBS. Add lysate in a ratio of 150 ul per well of the six-well plate. Use a micropipette to blend a few times to make full contact with the cell lysate. Protein concentration was measured. Equal amounts of protein (30 ug) were loaded for electrophoresis and transferred onto PVDF membranes. Blots were blocked in 1×TBST with 5% milk before incubation overnight at 4 °C with 1:1000 ATP6AP2 (ab64957), 1:5000 GAPDH (protein tech,10494-1-AP), 1:1000 caspase-1p20/p10 polyclonal antibody (protein tech,22915-1-AP), 1:1000 IL-1β (affinity, AF5130), 1:1000 NLRP3 (protein tech,19771-1-AP). Blots were washed with 1×TBST, 1:10000 secondary antibody conjugated with HRP was incubated at room temperature for 1 h, washed with 1×TBST, ECL substrate (Immobilon ECL, WBULS0500) was used for detecting, membranes were visualized by GE AI600 via chemiluminescence and analyzed with Image J.

### Quantitative RT-PCR

Total RNA from tissue and culture cells was isolated using TRIZOL reagent. RNA was reverse transcribed with Prime Script RT Reagent Kit (Takara; RR037A). qRT-PCR was performed via Light Cycler 480 SYBR Green I Master (Roche; 04887352001). Expression levels of RNA were normalized to GAPDH. Primers for mouse gene expression are shown in the table (Supplementary Table1). Relative quantitation was determined by the 2^—ΔΔCT^ method.

### ELISA

The mouse blood was collected in a clean test tube, coagulated at room temperature, and centrifuged at 2000 × *g* for 15 min. The serum was collected and stored at −80 °C after dividing. For cell culture supernatant, they were centrifuged to remove the precipitate and analyzed immediately. The biomarkers IL-1β ((EK0394, BOSTER)) and TNF-α (MTA00b, Quantikine^TM^ mouse TNF-a Immunoassay) was quantified by Mouse ELISA kit.

### Transverse aortic constriction surgery (TAC)

TAC surgery was performed on anesthetized mice 8–12 weeks of age as described previously [66]. Mice were randomized into either a sham group or a TAC group by a random number table. Briefly, the operating field was disinfected with 75% alcohol, and surgical tools were sterilized. The heating pad was maintained at 37 ± 1 °C. 2% isoflurane was used to maintain anesthetization. Mice were placed in the supine position, shaved fur with hair clippers. The transverse aortic arch was visualized through median sternotomy. A 27-gauge blunt needle was used to yield a 0.4 mm narrow in diameter, 7.0 silk suture ligature was performed. 6.0 silk suture ligature was used to close the rib cage and skin. Sham-operated mice performed the same operation except for constriction of the aortic arch. Warm-maintaining measure after the operation is conducive to recovery.

### Ultrasound echocardiography

Ultrasound echocardiography (Vevo 2100, VisualSonics, Toronto, Canada) with a transducer frequency of 40 HZ was used. Five percent isoflurane was inhaled to induce anesthetization and 1.5% isoflurane for anesthesia maintenance during the operation. The heart rate of the mice was maintained between 450 and 500 beats/min. The heart structure was recorded by M-mode ultrasound at the papillary muscle section. Analysis was performed using the software Vevo2100. Parameters including LVEDD, LVESD, LVID, LVEF, LVFS (%) were used to evaluate the changes in cardiac function by investigators blind to mice genotype.

### Immunohistochemistry and immunofluorescence microscopy analysis

Ventricles were fixed in 4% paraformaldehyde (PFA) for 24 h, dehydrated by gradient alcohol, transparent by xylene, and embedded in paraffin. Tissues were cut into sections of 5 um thickness, deparaffinization, rehydrated to complete the removal of paraffin. Hematoxylin and eosin (H&E) staining were used for histological analysis. Fibrillar collagen was detected with Masson’s trichrome. Additionally, sections were blocked protein and endogenous enzymes, antigen retrieval, primary for ATP6AP2 (1:200, Abcam, ab64957), NLRP3 (1:200, protein tech,19771-1-AP), Caspase1 (1:200, protein tech, 22915-1-AP), IL-1β (1:200, affinity, AF5130), IL-18 (protein tech,10663-1-AP) was performed for 24 h at 4 °C. After washing with TBST, sections were incubated with HRP conjugated goat-rabbit IgG or AlexaFluor-conjugated secondary antibody (Abcam, ab150077, ab150079). Analysis was performed by Image-J software for quantification of fibrosis by investigators blind to mice genotype.

### Reactive oxygen species (ROS) measurement-DCFDA

Isolated NRVMs were suspended in DMEM completed medium and exposed to phenylephrine after transfection with adenovirus-mediated Scramble (sh-Scr) or ATP6AP2 (sh-ATP6AP2) and stimulated with MCC950 (5 uM). DCFDA/H2DCFDA-Cellular ROS Assay Kit (ab113851, Abcam) was used to quantitatively assess reactive oxygen species in live-cell samples according to the manufacturer’s instructions.

### Mitochondrial ROS detection

MitoSOX Mitochondrial Superoxide Indicator (40778ES50, YEASEN) was used to assess mitochondrial ROS. NRVMs were seeded on cover slides of 12-well plates and transfected with ShR-ATP6AP2 or shR-Scr virus vector for 24 h at MOI = 50 on the 4th day. The medium was changed to a complete culture medium and continued for 48 h of PE stimulation or DMSO control. Then added 37 °C pretreated MitoSOX (500 nM) and incubated for 20 min. After PBS cleaning, the nuclei were stained with DAPI. Imaging with fluorescence microscopy (Ex = 579NM, Em = 599 nm).

### Ad-mCherry-GFP-LC3B adenoviral vector to detect autophagy

Ad-mCherry-GFP-LC3B (C3011, Beyotime) was used to detect the autophagy flow of infected cells according to the manufacturer’s instructions. Isolated NRVMs were plated in 24 orifice plates. Sh-Scr, sh-ATP6AP2(MOI = 50), and Ad-mCherry-GFP-LC3B adenoviral vector (MOI = 30) infection were performed after NRVMs reached 70% confluence. About 24 h after infection, the culture medium containing the virus was removed, 2 ml fresh complete culture medium was added to each well, and the growth status and fluorescent protein expression of cells were observed after 24 h, 48 h further culture. a fluorescence microscope was used to observe LC3B fluorescence changes.

### Flow cytometry-TMRE

NVRM cells were transfected with sh-ATP6AP2 or sh-Scr, pretreated with mito-tempo, and then stimulated with phenylephrine (100 umol/L) or DMSO for 6 h. TMRE-Mitochondrial Membrane Potential Assay Kit (ab113852, Abcam) was used to quantify changes in mitochondrial membrane potential in live cells by flow cytometry according to the manufacturer’s instructions.

### ICC/IF

Cell survival and death were assessed with fluorescent dyes (SYTO ^TM^ Orange s11368, and SYTO^TM^ Green, S7578). To show that phenylephrine can induce oxidative stress injury and ShR-ATP6AP2 can aggravate phenylephrine-induced oxidative stress injury, NVCM was labeled with double fluorescent dye to distinguish live from dead cells. After brief incubation with SYTOX Orange nucleic acid stain, the nucleic acids of dead cells fluoresce bright orange when excited with the 547 nm. SYTO Green is a cell-permeant nucleic acid stain that preferentially labels live-cell nuclei. The nucleic acids of live cells fluoresce bright green when excited with the 517 nm. NRVMs were infected with sh-Scr or sh-ATP6AP2, then phenylephrine stimulation for 36 h, after that incubated with SYTOX Orange and SYTOX Green at a concentration of 5 μM for 30 min at 37 °C. NRVMs were visualized by a fluorescence microscope.

### Lysosomal PH measurement

lysosomal PH was quantitatively detected by Lyso-Tracker Red (C1046, Beyotime) according to the manufacturer’s instruction. Isolated NRVMs were plated in 24 orifice plates. ShR-Scr, shR-ATP6AP2(MOI = 50) for 24 h. The cell culture medium was removed, and 37 °C preincubated lyso-Tracker Red staining solution was added, and the cells were co-incubated at 37 °C for 20 min. Lysotracker Red staining solution was removed, and a fresh cell culture solution was added. They were then observed under a fluorescence microscope.

### TUNEL

TUNEL assay in tissue was performed according to the manufacturer’s directions (TUNEL Andy Fluor TM 488 Apoptosis Kit, A050, ABP biosciences).

### Cardio-restricted ATP6AP2 overexpression transgenic mice

Tg mice expressing ATP6AP2 (H11-CAG-LSL-ATP6AP2-polyA Cas9-KI) were generated under the direction of the murine -CAG promoter by using CRISPR/Cas9 technology (Supplementary Fig. 1). The brief process is as follows: ATP6AP2 sgRNA(5’-3’:CTGAGCCAACAGTGGTAGTA) was transcribed in vitro, donor vector was constructed. Cas9, sgRNA, and Donor were microinjected into the fertilized eggs of C57BL/6 J mice. Fertilized eggs were transplanted to obtain positive F0 mice, which were confirmed by PCR. ATP6AP2-Tg mice were backcrossed to C57BL/6 mice to generate F1 mice. Mice carrying floxed atp6ap2 alleles (ATP6AP2fl/fl) were crossed with mice Cre recombinase controlled by the alpha-MyHC promotor. Male transgenic mice were used for experiments. Littermates wild-type or male age-matched WT mice were used as controls.

### Statistical analysis

For continuous data of normally distributed variables, data are expressed as mean ± SEM. One-way ANOVA was followed by the Tukey post-hock test for multiple groups. All results in nonnormally distributed variables are presented as medians and quartiles. Comparisons of the two groups were accomplished using the Mann–Whitney test where appropriate. Experiments with >2 groups were compared by Kruskal–Wallis one-way ANOVA followed by Dunn post-hoc test. The sample size was calculated by PASS software. All statistics were calculated using GraphPad Prism 7 (GraphPad Software Inc). *P*-values < 0.05 were considered statistically significant.

## Supplementary information

supplemental material

Fig S1

Fig S2

Fig S3

reproducibility checklist

## Data Availability

Supplementary information is available at cell death discovery’s website.
