# Supplementary material for: ATP6AP2 knockdown in cardiomyocyte deteriorates heart function via compromising autophagic flux and NLRP3 inflammasome activation
Source: Cell Death Discov. 2022 Apr 4;8:161. doi: 10.1038/s41420-022-00967-w (PMC8980069; doi:10.1038/s41420-022-00967-w)
Supplement: Supplementary file 1 — supplemental material [file 41420_2022_967_MOESM1_ESM.docx]

**Supplemental Table. S1** list of primers

**Supplemental Table. S2** WT and Tg-ATP6AP2/PRR mice were subjected to TAC to induce heart failure. 4 weeks later, echocardiography was performed. Echocardiographic parameters of WT-Sham, WT-TAC, Tg-Sham, and Tg-TAC. LVIDd, diastolic left ventricular dimension; LVIDs, systolic left ventricular dimension; LVFS, fractional shortening, BW, bodyweight: HW, heart weight; LVW, left ventricular weight; Values shown are mean±SEM. ^*^P<0.05, ^**^P<0.01, significance vs WT-sham; ^#^P<0.05, ^##^P<0.01 significance vs Tg-sham; by one-way ANOVA.

**Supplemental Table. S3** Sh-Scr and sh-ATP6AP2/PRR mice were subjected to TAC to induce heart failure. 4 weeks later, echocardiography was performed. Echocardiographic parameters of Scr-Sham, Scr-TAC, shATP6AP2-Sham, and shATP6AP2-TAC. LVEDD, Left Ventricular End Diastolic Diameter; LVESD, Left Ventricular End Systolic Diameter; LVFS, fractional shortening; dP/dtmax, peak instantaneous rate of left ventricular pressure; dP/dtmin, peak instantaneous rate of decline in left ventricular pressure increase; LVEDP, LV end-diastolic pressure. ^*^P<0.05 vs Scr-sham group; **P <0.01vs Scr-sham; ^#^P<0.05 vs Scr-TAC by ANOVA

**Supplemental Fig. S1** Strategy used to generate a conditional Atp6ap2 allele. The genomic structure, targeting vector, and targeted allele are shown.

**Supplemental Fig. S2.** **(ACDE)**Representative echocardiography images show peak velocity and statistical analysis of aortic coarctation at different times after ultrasound examination of TAC. **(BFGH)**Representative immunohistochemical staining and quantitative analysis of Caspase1， IL-1β， IL-18 expression between different groups. (scale bar=20μm).

**Supplemental Fig. S3. （AB）**showed the knockdown efficiency of shR-ATP6AP2 in cardiac tissue. **(C)** indicated expression of ATP6AP2, LC3A/B, SQSTM1, NLRP3, pro-caspase1 after stimulation with phenylephrine for different time points.

**Supplement Table. S1: List of primers**

|  |  | | **forward 5'-3'** |  |  | **reverse 5'-3'** |  |  |
| --- | --- | --- | --- | --- | --- | --- | --- | --- |
| IL-1b | mus | 5'TGACAGTGATGAGAATGACCTGTCC-3' | | |  | 5'-TTGGAAGCAGCCCTTCATCT-3' | | |
| IL-6 | mus | 5'-GCTACCAAACTGGATATAATCAGGA-3' | | |  | 5'-CCAGGTAGCTATGGTACTCCAGAA-3' | | |
| TNF-a | mus | 5'-CGGAGTCCGGGCAGG-3' | | |  | 5'-GCTGGGTAGAGAATGGATGAA-3' | | |
| NPPA | mus | 5-GAAGATGCCGGTAGAAGATGAG-3' | | |  | 5-AGAGCCCTCAGTTTGC TTTTC-3' | | |
| NPPB | mus | 5-TGC TTT GGG CAG AAG ATA GA-3 | | |  | 5-CAG CCA GGA GGT CTT CCT AA-3' | | |
| MYH6 | mus | 5'-TCTGGATTGGTCTCCCAGC-3 | | |  | 5-GTCATTCTGTCACTCAAACTCTGG-3' | | |
| MYH7 | mus | 5-CTGAAAGCAGAAAGAGATTATC-3 | | |  | 5-TGGAGTTCTTCTCTTCTGGAG-3' | | |
| GAPDH | mus | 5'-TCATCCCTGCATCCACT-3' | | |  | 5'-TCCACGACGGACACATT-3' | | |
| GAPDH | RAT | 5’-CCGCATCTTCTTGTGCAGTG-3' | | |  | 5'-AGAAGGCAGCCCTGGTAAC-3' | | |
| IL-1b | RAT | 5'-CACACTAGCAGGTCGTCATCATC-3' | | |  | 5'-ATGAGAGCATCCAGCTTCAAATC-3' | | |
| il-18 | RAT | 5’-ACAGAGGATGAGACTGAGGCAC-3' | | |  | 5'-TATTCAGGCTGGGTCCTGTCAC-3' | | |
| IL-6  PRR  PRR | RAT  Mus  RAT | 5'-GCCCTTCAGGAACAGCTATGA-3'  5'-TTCTGAACTGCAAGTGCTGCAT-3'  5′-TCTGTTCTCAACTCGCTCC C-3 | | |  | 5'-TGTCAACAACATCAGTCCCAAGA-3'  5'-CTGCCAGCTCCAGTGAATACAAG-3'  5′-TCTCCATAACGCTTCCCAAG-3′ | | |

**Supplement Table.S2** Echocardiographic parameters of WT-Sham, WT-TAC, Tg-Sham and Tg-TAC

|  | WT-sham  (n=4) | WT-TAC  (n=4) | Tg-sham  (n=4) | Tg-TAC  (n=4) |
| --- | --- | --- | --- | --- |
| BW（g） | 25.3±0.3 | 22.5±0.37 | 24.9±0.43 | 23.85±0.78 |
| HW(mg) | 110.2±1.4 | 237.8±6.6^**^ | 109.7±1.8 | 241.7±7.9^##^ |
| LVW(mg) | 78.4±0.8 | 157.3±1.6^**^ | 79.6±1.5 | 160.3±0.89^##^ |
| Lung weight | 139.2±2.1 | 218.6±3.7^*^ | 131.6±2.5 | 209.7±4.1^#^ |
| Liver weight | 1057.8±36.9 | 1565.9±46.8^*^ | 1046.3±21.7 | 1496.8±35.6^#^ |
| LVW/BW | 3.13±0.06 | 4.75±1.33^*^ | 3.16±0.05 | 4.66±1.29^#^ |
| LVIDd | 2.75±0.02 | 3.85±0.09^*^ | 2.69±0.03 | 3.79±0.03^#^ |
| LVIDs | 1.46±0.02 | 2.13±0.02^*^ | 1.43±0.01 | 2.18±0.03^#^ |
| LVFS | 48.7±0.68 | 38.8±1.04^*^ | 47.4±0.52 | 37.5±0.97^#^ |

Echocardiographic parameters of WT-Sham, WT-TAC, Tg-Sham and Tg-TAC. LVIDd, diastolic left ventricular dimension; LVIDs, systolic left ventricular dimension; LVFS, fractional shortening, BW, body weight;. HW, heart weight; LVW, left ventricular weight;Values shown are mean±SD. ^*^P<0.05, ^**^P<0.01, significance vs WT-sham;^#^P<0.05, ^##^P<0.01 significance vs Tg-sham; by one way ANOVA.

**Table. S3** Echocardiographic parameters of Scr-Sham, Scr-TAC, shPRR-Sham and shPRR-TAC

|  | Scr-sham | shPRR-sham | Scr-TAC | shPRR-TAC |
| --- | --- | --- | --- | --- |
| LVEDD(mm) | 3.46±0.02 | 3.49±0.03 | 3.87±0.05 ** | 4.26±0.07 # |
| LVESD(mm) | 1.88±0.02 | 1.9±0.02 | 2.44±0.06 ** | 3.11±0.1 # |
| LVFS% | 45.6±0.72 | 45.4±0.68 | 37±0.97 ** | 27.1±1.14 # |
| LVEDP(mmHg) | 4.62±0.39 | 5.3±0.41 | 10.3±0.76 * | 17.7±0.73 # |
| dP/dtmax(mmHg/s) | 7578±483 | 7758±451 | 5958±402 * | 5123±498 |
| dP/dtmin(mmHg/s) | 7414±649 | 7085±569 | 5339±529 * | 4962±573 |
| HR（BMP） | 449±16 | 437±14 | 458±14 | 459±17 |

Echocardiographic parameters of Scr-Sham, Scr-TAC, shPRR-Sham and shPRR-TAC. LVEDD, Left Ventricular End Diastolic Diameter; LVESD, Left Ventricular End Systolic Diameter;LVFS, fractional shortening; dP/dtmax, peak instantaneous rate of left ventricular pressure; dP/dtmin, peak instantaneous rate of decline in left ventricular pressure increase; LVEDP,LV end-diastolic pressure. *P<0.05 vs Scr-sham group; **P <0.01vs Scr-sham; #: P<0.05 vs Scr-T.AC by ANOVA
