# Supplementary figures and images for: ATP6AP2 knockdown in cardiomyocyte deteriorates heart function via compromising autophagic flux and NLRP3 inflammasome activation
Source: Cell Death Discov. 2022 Apr 4;8:161. doi: 10.1038/s41420-022-00967-w (PMC8980069; doi:10.1038/s41420-022-00967-w)

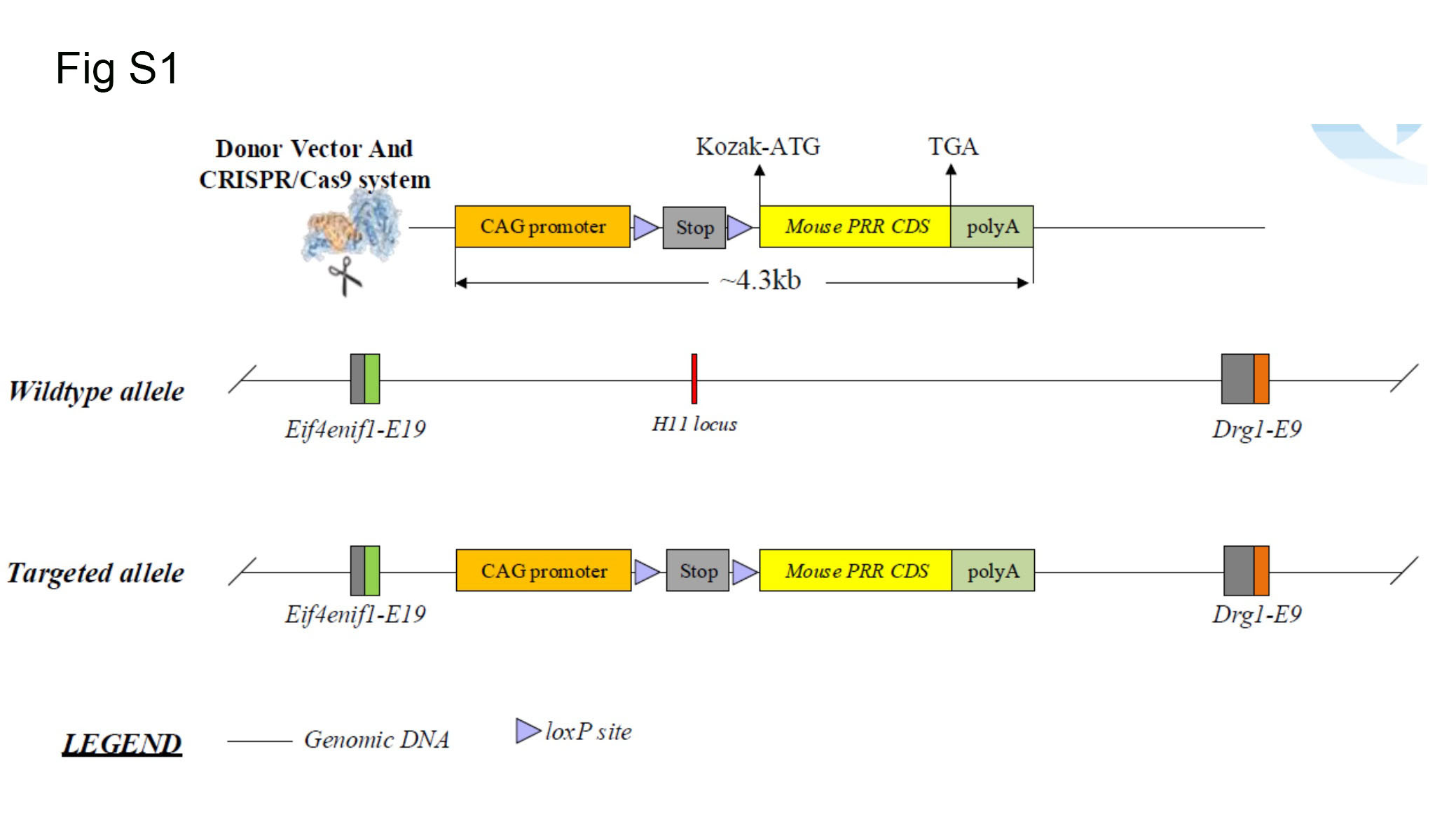

Supplement: Supplementary file 2 — Fig S1 [file 41420_2022_967_MOESM2_ESM.tif]

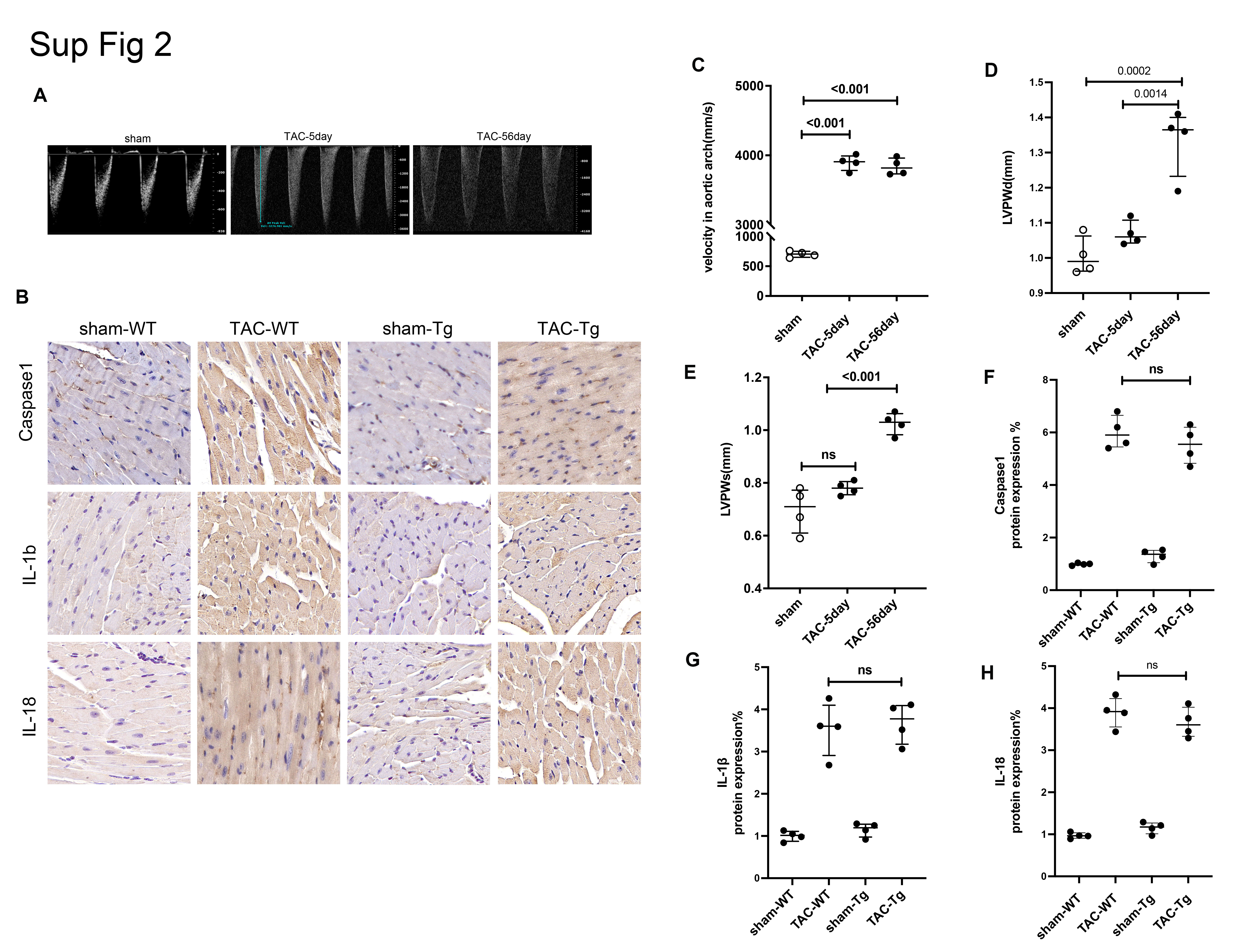

Supplement: Supplementary file 3 — Fig S2 [file 41420_2022_967_MOESM3_ESM.tif]

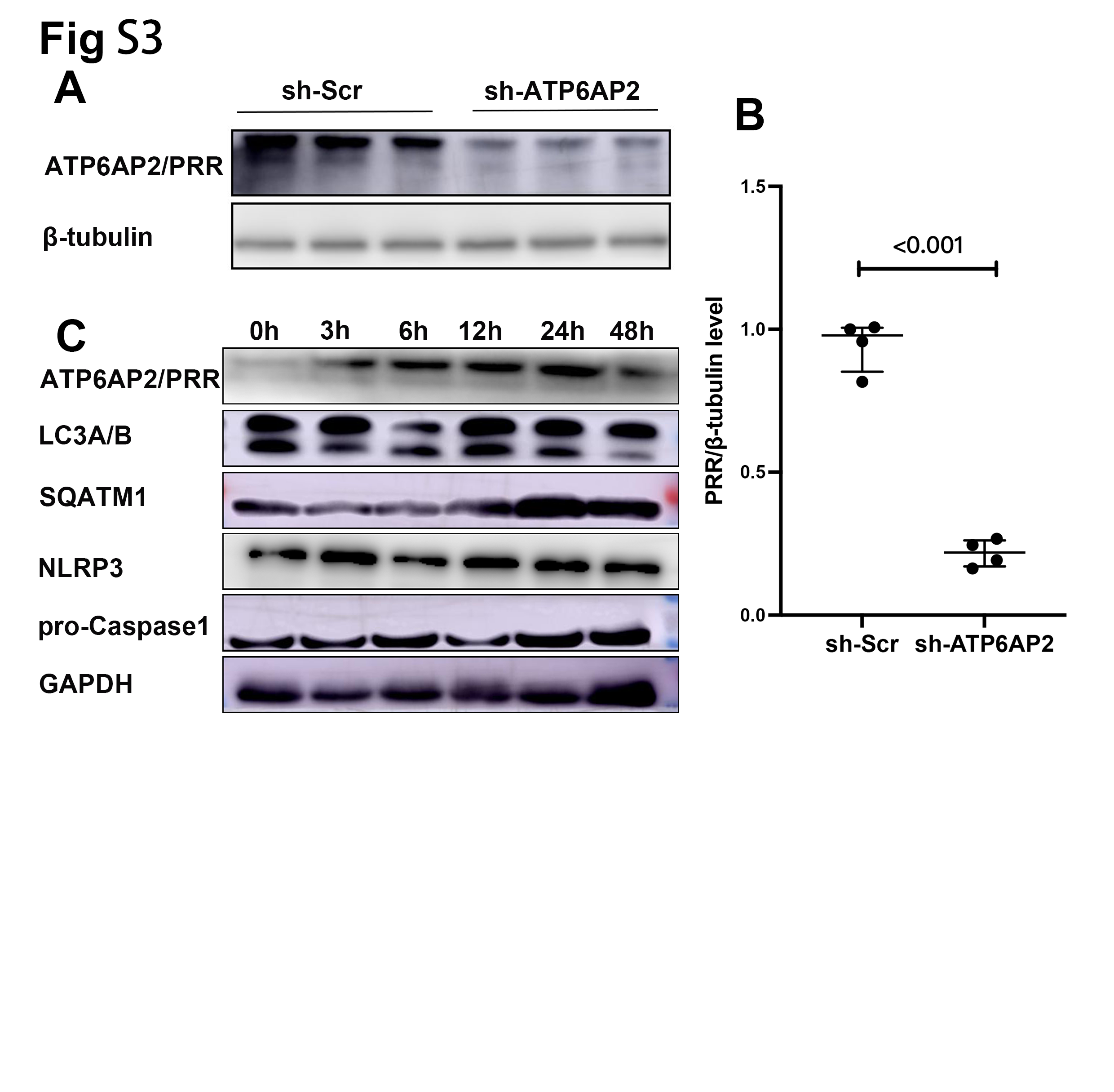

Supplement: Supplementary file 4 — Fig S3 [file 41420_2022_967_MOESM4_ESM.tif]
